# The impact of family planning on maternal mortality in Indonesia: what future contribution can be expected?

**DOI:** 10.1186/s12963-020-00245-w

**Published:** 2021-01-11

**Authors:** Budi Utomo, Purwa Kurnia Sucahya, Nohan Arum Romadlona, Annette Sachs Robertson, Riznawaty Imma Aryanty, Robert Joseph Magnani

**Affiliations:** 1 Department of Population and Biostatistics, Faculty of Public Health, Universitas Indonesia, Depok, Indonesia; 2 Center for Health Research, Universitas Indonesia, Depok, Indonesia; 3 UNFPA Asia and the Pacific Regional Office, Bangkok, Thailand; 4 UNFPA Indonesia, Jakarta, Indonesia; 5 Faculty of Public Health, Universitas Indonesia, Depok, Indonesia

**Keywords:** Family planning, Maternal mortality, Indonesia

## Abstract

**Background:**

Although efforts to reduce high maternal mortality in countries such as Indonesia tend to focus on addressing health risks among pregnant women, family planning has been shown globally to reduce maternal mortality by reducing both total and higher-risk pregnancies. This article assesses past contributions of family planning to the reduction of maternal mortality in Indonesia and the potential future contribution toward achieving the 2030 SDG maternal mortality goal.

**Methods:**

The study takes advantage of data from long series of population censuses and large-scale surveys that are available in few other low- and middle-income countries. We use the decomposition method suggested by (Matern Child Health J, 16:456–463, 2012) and regression-based policy simulations to estimate the number of maternal deaths averted during 1970–2017 due to contraceptive use and project potential future contributions to the year 2030.

**Results:**

It is estimated that between 523,885 and 663,146 maternal deaths were averted from 1970 to 2017 due to contraceptive use, a 37.5–43.1% reduction. If the contraceptive prevalence rate (CPR) were to rise from 63% in 2017 to 70% in 2030 and unmet need for family planning were to fall to from 10 to 7%, an additional 34,621–37,186 maternal deaths would be averted, an 18.9–20.0% reduction. A 2030 CPR of 75% and unmet need for family planning of 5% would result in 51,971–54,536 maternal deaths being averted, a 28.4–29.4% reduction. However, the CPR growth rate would have to nearly double the 2000–2017 rate to reach 70% CPR by 2030 and more than triple to reach 75%. Achieving the most ambitious target would still leave the maternal mortality ratio at 125 in 2030 without corresponding improvements in maternal health services.

**Conclusions:**

Although substantial reductions in maternal mortality between 1970 and 2017 can be attributed to contraceptive use and further contributions to the year 2030 are probable, smaller contributions are likely due to the already relatively high CPR and the challenges that must be overcome to move the CPR significantly higher. The ability of Indonesia to reach the 2030 SDG maternal mortality target of 70 maternal deaths per 100,000 live births will depend primarily upon health system effectiveness in addressing health risks to women once they are pregnant.

## Introduction

Globally, both the number of maternal deaths and the rate at which maternal deaths occur have declined dramatically since 1990 [3, 29]. Despite adopting comprehensive safe-motherhood policy measures, maternal mortality in Indonesia remains unacceptably high. Various methods of estimating maternal mortality in Indonesia during the past decade have consistently produced maternal mortality ratios (MMR) between 200 and 350 maternal deaths per 100,000 live births [10, 17]. The current official Government of Indonesia estimate of the maternal mortality ratio based on the 2015 Inter-Censal Population Survey (SUPAS) is 305 maternal deaths per 100,000 live births [19]. The global Maternal Mortality Estimation Inter-Agency Group, using a different methodology than the Government of Indonesia, put the MMR at 126 per 100,000 live births in 2015 [29]. Irrespective of which estimate is used, the Indonesian MMR is high. By way of comparison, the estimated MMRs in 2017 in Association of Southeast Asian Nations (ASEAN) comparator nations were Thailand (20), Brunei (23), Malaysia (40), Vietnam (54), and Philippines (114) [30]. Most maternal deaths are preventable, as the health care solutions to prevent or manage complications are well known [29].

A global consensus has emerged as to core strategies to reducing maternal mortality. These consist of (1) family planning with related reproductive health services, (2) skilled care during pregnancy and childbirth, (3) timely emergency obstetric care, and (4) immediate postnatal care [28]. In contrast with the other three interventions, which focus on reducing risk among women who are or recently were pregnant, family planning programs reduce maternal mortality by (1) reducing the number of pregnancies that occur and (2) reducing the proportion of pregnancies that are deemed to be “higher-risk” [16]. Fewer pregnancies translate into a reduction in the number of times that women are exposed to the risk of maternity-related mortality, an impact that compounds over time as fewer births yields successive generations of smaller cohorts of women of reproductive age. Contraceptive use is a key direct determinant of fertility reduction [5, 9, 23], the other “proximate determinants” being marriage/sexual exposure, postpartum infecundability, and induced abortion [6]. Contraceptive use also lowers the risk of maternal mortality per birth, as measured by the MMR, by preventing high-risk births, that is, births to women who are “too young” or “too old,” birth intervals that are “too close,” and high-parity births (i.e., “too many”) [8, 23]. Family planning has been estimated to have reduced maternal mortality levels in various countries by magnitudes ranging from 6 to 60% [1, 7] − 44% globally [16], as well as lowering infant mortality and abortion rates, especially unsafe abortions [13, 26]. Mbizvo and Burke [14] estimate that globally family planning could prevent up to 30% of maternal deaths going forward.

Indonesia has been among the global leaders in family planning. The success of the national family planning program is evidenced by the sharp increase in the contraceptive prevalence rate (CPR) among married women from 8% in the early 1970s to 60% in 2002, while during the same time period the total fertility rate (TFR) was reduced by nearly one-half from 5.0 to 2.6 [18, 22]. Although the rate of growth in contraceptive use has slowed since the early of 2000s, CPR reached 63% in 2017 and the TFR fell to 2.3 [20].

As the Government of Indonesia struggles to reduce stubbornly high rates of maternal mortality, the research reported in this article sought to provide a clearer understanding as to the impact that contraceptive use had had on maternal mortality in the country over a 35-year period and the likely magnitude of future contributions. Having scientifically sound assessments of future contributions is important for national policy and resource allocation decisions given the already relatively high level of contraceptive use and the diminishing rate of growth in contraceptive prevalence over the past two decades. We took advantage of long series of population census, large-scale surveys, and other data that are available in Indonesia but in few other low-and-middle-income countries to pursue these research objectives.

## Materials and methods

**Fig. 1:**
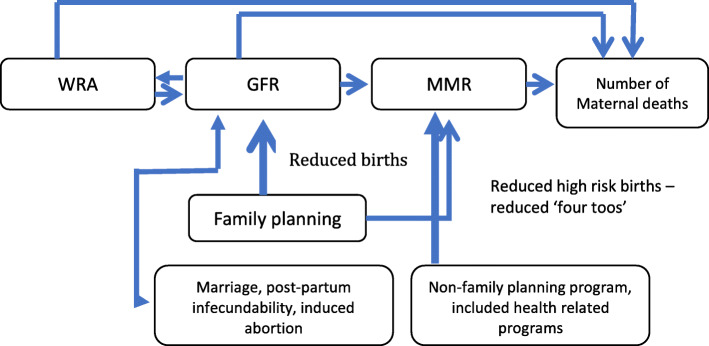
Analytic framework—how family planning reduces maternal deaths

The basic methodologies used in the study consisted of a modified version of the decomposition method suggested by Ross and Blanc [16] along with policy simulations. Ross and Blanc show that the number of maternal deaths in any given year can be decomposed into three statistical components: the number of women of reproductive age (WRA), the general fertility rate (GFR), and the maternal mortality ratio (MMR) (see Fig. 1). That is:

$$ \mathrm{MD}=\mathrm{WRA}\ast \mathrm{GFR}\ast \mathrm{MMR} $$

where:

MD = number of maternal deaths during a specified year

WRA = number of women at reproductive age at mid-point of a specified year

GFR = general fertility rate, defined as number of births during a specified year divided by number of WRA at the mid-point of a specified year

MMR = maternal mortality ratio in the specified year, defined as number of maternal deaths divided by number of live births during the same year

Data on WRA, GFR, and MMR for the years 1970 to 2017 needed for the maternal death decomposition analysis were gathered from multiple data sources (see Annex 1 for a full listing of data sources). Population data by age and gender were extracted from Population Censuses (1971, 1980, 1990, 2000, and 2010) and Inter-Censal Population Surveys (1985, 1995, 2005, and 2015). Annual, smoothed values during the 1970–2017 period were obtained using least-squares polynomial regressions. A similar process was followed in deriving annual figures of GFR and MMR, with fertility and maternal mortality data coming from a variety of surveys and other sources (see Annex 1).

To quantify the contributions of contraceptive use to declining maternal mortality, we ran a series of policy simulations in which we compared the results of the basic decomposition results against selected “counterfactual” scenarios. To estimate the impact of contraception on maternal mortality during the 1970–2017 period, the “baseline” decomposition results were compared against a counterfactual projection assuming no change in the contraceptive prevalence rate (CPR) between 1970 and 2017. The potential future impact of contraceptive use on maternal mortality was then estimated by comparing alternative scenarios with regard to the magnitude of CPR growth from 2017 to 2030. The first scenario assessed the impact of an increase in the contraceptive prevalence rate (CPR) from 63% in 2017 to 70% in the year 2030 and a decline in unmet need for family planning from 10% in 2017 to 7% in 2030. In the second scenario, CPR was assumed to increase from 63% in 2017 to 75% in 2030 and unmet need for family planning to decline from 10% in 2017 to 5% in 2030. The WRA figures used in the analyses were derived from Statistics Indonesia population projections to the year 2030 [18, 22]. Annual GFR and MMR values for the projections for the alternative scenarios were estimated using a regression approach, the details of which are documented along with the presentation of results.

In undertaking the analyses, we made two refinements to the basic decomposition method of Ross and Blanc [16]. One limitation of this decomposition approach, which was recognized by the authors, is that changes in GFR are not only the result of changes in contraceptive use but also of changes in other “proximate determinants” of fertility, the most prominent of which are marriage, postpartum infecundability, and abortion [4, 6]. To address this issue, we examined trends in the main non-contraception proximate determinants of fertility over the 1970–2017 period and, based upon these data and the published literature on this topic, derived an estimate of the percentage of the change in GFR that was attributable to contraceptive use. We undertook sensitivity analyses to assess the implications of uncertainty regarding this parameter on subsequent results. Further details are provided along with the presentation of results.

Second, as the global literature is clear that family planning affects the MMR by reducing the number of high-risk pregnancies/births as well as by reducing the number of women becoming pregnant, the observed MMR data will include these high-risk pregnancy/birth effects and it is thus necessary to account for this effect in order to correctly quantify the impact of contraceptive use in the policy simulations. To address this issue, we examined changes in age-specific fertility rates, parity, and lengths of closed birth intervals from 1970 to 2017, and as above based upon these data and the published literature, we derived an estimate of what the observed MMRs would have been in the absence of declines in the CPR. A sensitivity analyses was also undertaken for this sub-analysis to assess the implications of uncertainty on subsequent results. Further details are provided along with the presentation of study findings.

## Results

### Baseline decomposition results

**Fig. 2:**
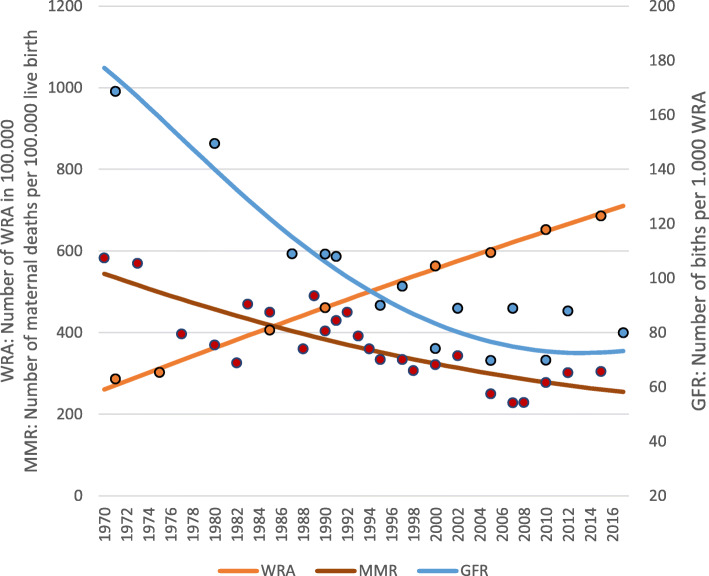
Trends of WRA, GFR, and MMR, Indonesia 1970–2017. Note: the dots are observed data points, while the lines are smoothed regression estimates

The annual estimates of the maternal death decomposition components (WRA, GFR, and MMR) developed for the period of 1970–2017 are displayed in condensed form for selected years in Table 1. Despite the number of WRA having almost tripled during this reference period, the annual number of births varied within a relatively narrow range (4.6 to 5.2 million) due to a 64% reduction in the GFR. The analysis used the best-fitting quadratic regression *y* = 0.0697*x*^2^ − 9.5793*x* + 553.86 (R square = 0.6656) to smooth the trend of reported MMR estimates during the period. Based upon these procedures, it is estimated that the MMR fell from 544 in 1970 to under 300 by 2005 and has since been declining at a slower pace. This resulted in a 43.7% reduction in the annual number of maternal deaths from just over 25,000 in the early 1970s to just over 13,000 in 2017. The maternal mortality rate, which is a measure of the risk of maternal deaths per 1000 WRA, fell by 82% during this same period. The trends in WRA, GFR, and MMR are shown visually in Fig. 2.

**Table 1:** Maternal death decomposition results, Indonesia 1970–2017

Years	WRA	GFR	Births	Maternal mortality ratio	Maternal deaths	Maternal mortality rate
1970	26,042,390	177	4,617,379	544	25,135	0.97
1975	31,200,951	159	4,966,205	499	24,776	0.79
1980	36,270,530	140	5,075,444	457	23,191	0.64
1985	41,251.126	122	5,026,855	418	21,034	0.51
1990	46,142,740	106	4,894,523	383	18,767	0.41
1995	50,945,371	93	4,744,862	352	16,698	0.33
2000	55,659,020	83	4,636,614	324	15,017	0.27
2005	60,283,686	77	4,620,847	299	13,832	0.23
2010	64,819,370	73	4,740,958	278	13,193	0.20
2015	69,266,071	73	5,032,670	261	13,120	0.19
2017	71,019,837	73	5,203,944	255	13,251	0.19

Births =WRA × GFR; Maternal deaths (MD) = WRA × GFR × MMR; MMRate = MD/WRA

### Contraception and other proximate determinants of fertility decline

**Fig. 3:**
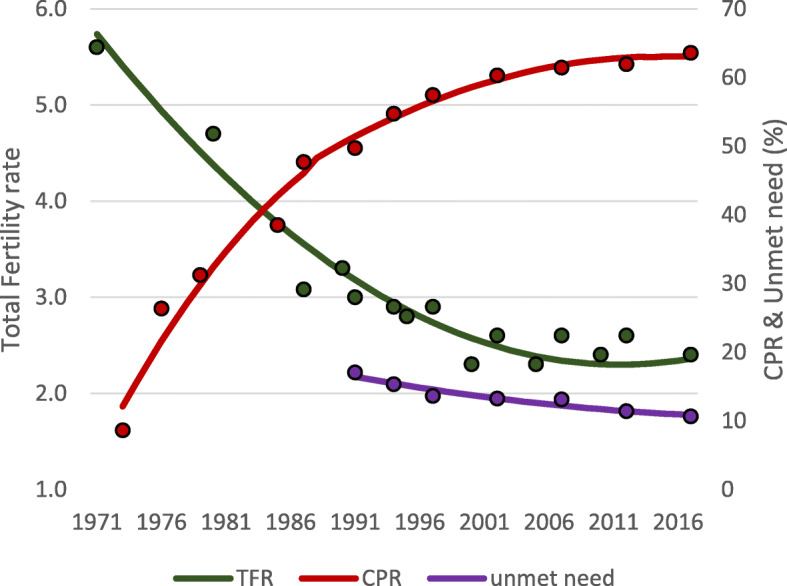
Trends of CPR, UNFP, and TFR, Indonesia 1971–2017

To what extent can the decline in the GFR be attributed to increases in contraceptive use? As shown in Fig. 3, since the start of the national family planning program in the early of 1970s, the CPR increased sharply to over 40% in the early 1990s and to 63% in 2017, while TFR declined sharply from just under 6 in the early 1970s to 2.3 in 2017, although the rates of decline have slowed considerably since the early 2000s. Unmet need for family planning (UNFP) declined from 17.0% in 1991 to 10.6% in 2017.

Trends in the major proximate determinants of fertility other than contraception are shown in Table 2. With regard to marriage, singulate mean age at first marriage increased rather substantially from 19.1 years in 1987 to 22.4 years in 2017, while the proportion of women ages 45 and above who never married also increased slightly. These trends would both have had a dampening effect on fertility. The median duration of exclusive breastfeeding increased since 1995, while the median duration of any breastfeeding declined by a comparable amount. Given that the median length of exclusive breastfeeding in Indonesia remains relatively short, these trends are unlikely to have had a major influence on fertility trends over the past 20 or so years. We were precluded from including abortion in the analysis due to a lack of reliable data.

**Table 2:** Trends in proximate determinants of fertility other than contraception, 1970–2017

Year	Singulate mean age at first marriage	% never married	Median duration of exclusive breastfeeding	Median duration of any breastfeeding
**1970**	19.1	1.5	na	na
**1975**	19.5	1.9	na	na
**1980**	20.3	2.2	na	na
**1985**	20.9	2.5	na	na
**1990**	21.5	2.7	na	na
**1995**	21.9	2.8	1.8	23.0
**2000**	22.2	2.9	1.5	22.4
**2005**	22.3	2.9	1.0	21.9
**2010**	22.3	2.9	1.1	21.6
**2015**	22.3	2.7	2.6	21.4
**2017**	22.4	2.7	3.7	21.3

Earlier data from low- and middle-income countries indicated that differences in contraceptive prevalence explained about 90% of the variation in fertility rates across countries [15]. Based on the available Indonesian data and global evidence, we conservatively assumed that contraceptive use was responsible for 80% of reduced fertility during the 1970–2017 period in the policy simulations. However, especially given the lack of information on abortion levels or trends, we undertook sensitivity analyses in the policy simulations in which we assessed the consequences of this parameter being either too high or too low—specifically, 85% and 75%.

### Contribution of family planning to reducing high-risk births

To assess family planning contributions to reducing high-risk births, we examined trends in factors that have been linked with high-risk births in the literature. These factors included unwanted births and births from pregnancies that were “too young, too old, too many or too close.” Although it is difficult to isolate the specific effects of wanted status on maternal mortality from the effects of other factors because a significant number of unwanted pregnancies occur to women who are at also risk for other reasons (e.g., age, parity, time since last birth), the literature suggests a strong link between unwanted status and abortion, which is clearly associated with maternal mortality risk [23, 26]. An analysis of the planning status of recent births from the series of seven (7) DHS surveys undertaken in Indonesia indicates an increase in proportion of recent births that were “wanted then” from 77.4% in 1987 to 84.0% in 2017.

More substantial changes over time are observed with regard to age, parity, and birth interval risks (Table 3). With regard to age risk, the percent declines in age-specific fertility rates among young (ages 15–19 years) and older women (ages 40–49 years) between 1970 and 2017 exceeded that for women in the “safe” ages, and by a considerable margin. The proportion of births resulting from pregnancies that were “too many” and/or “too close” have also declined significantly. The proportion of last births that were parity four or above declined from 35% in 1987 to 12% in 2017, while the median of length of the interval between the last two births lengthened from 38 months in 1991 to 65 months in 2017. Based on the literature and the available Indonesian data, we used an estimate of 15% contribution of contraceptive use to the reduction of the MMR via reduction in numbers of high-risk births in our policy simulations. Sensitivity analyses were undertaken in connection with the policy simulations in which we assessed the consequences of this parameter instead being 20% and 10%, respectively.

**Table 3:** Age-specific fertility rates and total fertility rates, Indonesia 1970–2017

Year	Age group							TFR
	15–19	20–24	25–29	30–34	35–39	40–44	45–49	
1970	153	291	276	216	124	56	18	5.7
1975	128	254	242	186	109	48	15	4.9
1980	106	222	213	161	95	40	12	4.3
1985	87	194	188	140	84	34	10	3.7
1990	72	171	167	123	75	29	8	3.3
1995	59	151	151	110	68	25	6	2.9
2000	50	136	140	102	63	22	5	2.6
2005	44	125	133	99	60	20	4	2.5
2010	41	119	131	100	60	19	4	2.5
2015	42	117	133	105	61	19	4	2.4
2017	43	117	135	108	62	20	4	2.4
% decline 1970–2017	71.9	59.8	51.1	50.0	50.0	64.3	77.8	

### Family planning and maternal deaths averted

To assess the impact of contraceptive use on the number of maternal deaths, we compared the baseline decomposition analysis results (see Table 1) against a counterfactual projection that assumed no changes in CPR from 1970 onward. As described above, we simulated the impact of contraception on fertility by reducing the projected decline in annual GFRs in the counterfactual projection by 20% consistent with the above estimate that contraception accounted for 80% of changes in fertility and other proximate determinants the remaining 20%. Similarly, we adjusted the projected decline in the maternal mortality ratio (MMR) by 15% to reflect the estimated contribution to MMR reduction via the reduction in numbers of high-risk births. Tables 4 and 5 present the results of these policy simulations.

**Table 4:** Estimated number of births averted by the family planning, Indonesia 1970–2017

Year	Births Baseline	Births S1	No. births averted	% Births averted
**1970–1984**	74,353,916	85,521,342	11,167,427	13.1
**1985–1999**	72,537,644	117,138,797	44,601,154	38.1
**2000–2014**	70,601,092	147,446,679	76,845,587	52.1
**2015–2017**	15,350,822	33,265,359	17,914,537	53.9
**1970–2017**	232,843,474	383,372,178	150,528,704	39.3

Baseline—observed, smoothed data; S1 = scenario 1 of no family planning program—see text for assumptions

**Table 5:** Estimated number of maternal deaths averted by the family planning, 1970–2017

Year	WRA Base	WRA S1	GFR base	GFR S1	MMR Base	MMR S1	MD Base	MD S1	MDA	% MDA
**1970**	26,042,390	26,042,390	177	177	544	544	25,135	25,135	-	-
**1975**	31,200,951	31,200,951	159	174	499	506	24,776	27,404	2628	9.6
**1980**	36,270,530	36,270,530	140	170	457	470	23,191	28,953	5762	19.9
**1985**	41,251,126	41,251,126	122	166	418	437	21,034	29,985	8951	29.9
**1990**	46,142,740	46,267,274	106	163	383	408	18,767	30,748	11,981	39.0
**1995**	50,945,371	51,192,442	93	160	352	381	16,698	31,280	14,583	46.6
**2000**	55,659,020	56,026,641	83	159	324	357	15,017	31,699	16,682	52.6
**2005**	60,283,686	60,769,875	77	157	299	336	13,832	32,101	18,269	56.9
**2010**	64,819,370	65,422,149	73	156	278	318	13,193	32,571	19,379	59.5
**2015**	69,266,071	69,983,466	73	156	261	303	13,120	33,186	20,066	60.5
**2017**	71,019,837	71,782,525	73	156	255	298	13,251	33,488	20,236	60.4
**1970–1984**							358,424	417,08	59,484	14.2
**1985–1999**							270,128	463,704	193,576	41.7
**2000–2014**							205,752	484,704	278,952	57.6
**2015–2017**							39,546	100,006	60,460	60.5
**1970–2017**							873,850	1,466,322	592,472	40.4

Table 4 presents estimates of births averted during the 1970–2017 period and selected sub-periods therein. Comparing the estimates from the observed, smoothed data (referred to as the “Baseline”) and the scenario with no change in CPR after 1970 (scenario 1), it is estimated that the Indonesian national family planning program averted 150.5 million births during 1970–2017, a 40.7% averted. The majority of births averted, 94.8 million, occurred after the year 2000 when CPR had already surpassed 50%.

The estimated impact of these averted births on numbers of maternal deaths is shown in Table 5. It is estimated that the Indonesia national family planning program averted 592,472 maternal deaths between 1970 and 2017, a 40.4% reduction. Fifty-seven percent of the maternal deaths averted occurred after the year 2000. As may be observed, this is due to a combination of (1) fewer WRA after 1990 as a result of earlier declines in fertility, (2) fewer women being exposed to the risk of maternal mortality due to not becoming pregnant (i.e. a lower GFR), and (3) a reduced likelihood of dying once pregnant due to a reduction in the number of high-risk births in addition to improvements in maternal health services. Sensitivity analyses indicated that if contraceptive use were to have accounted for 85% of fertility decline instead of 80% and the reduction in high-risk births to have accounted for 20% impact on the MMR instead of 15% as in the above projection, the number of maternal deaths averted from 1970 to 2017 would have been 663,146 instead of 592,472, a reduction of 43.1% instead of 40.4%. Less aggressive assumptions (contraceptive use accounting for only 75% of fertility decline and having only a 10% impact on the MMR) resulted in an estimate of the number of maternal deaths averted during 1970–2017 of 523,885, a reduction of 37.5%.

### Potential future contributions

Given that the CPR in Indonesia reached 63% in 2017, what is the potential magnitude of further family planning program contributions to reducing maternal deaths? To address this question, we ran two simulations with different assumptions as to future levels of CPR and unmet need for family planning (UNFP) to year 2030. The target CPR in 2030 was set at 70% in scenario 2, and 75% in scenario 3. UNFP was assumed to decline from 10% in 2017 to 7% in 2030 in scenario 2 and 5% in scenario 3. Annual values of WRA were extracted from Statistics Indonesia population projections to the year 2030. As the projection period is only 14 years, too short a period time for scenario differences in fertility to affect relative numbers of WRA, the WRA values were the same in the baseline and two alternative scenarios. Baseline annual values of GFR and MMR were calculated assuming no further increases in CPR from 2017 onward.

The projected annual GFR values from 2017 to 2030 under scenarios 2 and 3 were estimated via fitted quadratic regressions of the form:

$$ {\mathrm{GFR}}_i=a+b{\mathrm{CPR}}_i+c{{\mathrm{CPR}}_i}^2+{e}_{\mathrm{i}} $$

where:

$$ {\mathrm{GFR}}_i=\mathrm{estimated}\ \mathrm{GFR}\ \mathrm{for}\ \mathrm{year}\ \mathrm{i}, $$

$$ {\mathrm{CPR}}_i=\mathrm{projected}\ \mathrm{CPR}\ \mathrm{for}\ \mathrm{year}\ \mathrm{i}, $$

*a*, *b*, and *c* are regression coefficients estimated from the CPR and GFR data from the years 1997–2017

$$ {e}_{\mathrm{i}}=\mathrm{an}\ \mathrm{error}\ \mathrm{term}\ \mathrm{assumed}\ \mathrm{to}\ \mathrm{uncorrelated}\ \mathrm{with}\ \mathrm{CPR}. $$

The “diagnostics” for the regressions for the 1997–2017 period, GFR_*i*_ = 580,335 + (− 14,188*CPR_i_) + (0.097*CPR_i_^2^), indicated an *R*^2^ of 0.995, and a *X*^2^ goodness of fit of 0.36 (*p* < .01).

The projected annual MMR values from 2017 to 2030 under the two scenarios were estimated via fitted linear regressions of the form:

$$ {\mathrm{MMR}}_i=a+b{\mathrm{CPR}}_i+c{\mathrm{UFP}}_i+{e}_i $$

where:

$$ {\mathrm{MMR}}_i=\mathrm{estimated}\ \mathrm{GFR}\ \mathrm{for}\ \mathrm{year}\;\mathrm{i}, $$

$$ {\mathrm{CPRi}}_i=\mathrm{projected}\;\mathrm{CPR}\kern0.17em \mathrm{for}\kern0.17em \mathrm{year}\;\mathrm{i}, $$

$$ {\mathrm{UNFP}}_i=\mathrm{projected}\ \mathrm{UNFP}\ \mathrm{for}\ \mathrm{year}\;\mathrm{i}, $$

*a*, *b*, and *c* are regression coefficients estimated from the CPR and UNFP GFR data from the years 1997–2017

$$ {e}_i=\mathrm{an}\ \mathrm{error}\ \mathrm{term}\ \mathrm{assumed}\ \mathrm{to}\ \mathrm{uncorrelated}\ \mathrm{with}\ \mathrm{CPR}\ \mathrm{and}\ \mathrm{UNFP} $$

The regression “diagnostics” for the 2017–2030 period, MMR_*i*_ = − 395,308 + (31,726*UNFP_i_) + (4824*CPR_i_), indicated an *R*^2^ of 0.999, and a *X*^2^ goodness of fit of 0.26 (*p* > 0.01).

The resulting projected GFRs and MMRs were then used in decomposition analyses similar to the analyses whose results are presented in Table 5 to estimate the annual number of maternal deaths that would be averted under the two alternative future scenarios against the baseline scenario of no further increase in CPR between 2017 and 2030. As in the above analyses, it was assumed that contraception was responsible for 80% of the decline in GFR and 15% of the reduction in the MMR (due to a reduction in the number of unwanted births). The results of the policy simulations are shown in Tables 6 and 7.

**Table 6:** Estimated number of deaths averted by the family planning, 2017–2030, under scenario 2

Year	WRA Base	WRA S2	GFR Base	GFR S2	MMR Base	MMR S2	MD Base	MD S2	MDA	% MDA
2017	71,019,837	71,019,837	73	73	255	255	13,251	13,251	-	-
2018	71,891,381	71,891,381	73	70	252	246	13,247	12,477	770	5.8
2019	72,759,365	72,759,365	73	70	249	239	13,241	12,107	1133	8.6
2020	73,623,790	73,623,790	73	69	247	233	13,231	11,745	1487	11.2
2021	74,484,656	74,484,656	73	68	244	226	13,220	11,388	1832	13.9
2022	75,341,963	75,341,963	73	67	241	219	13,206	11,038	2168	16.4
2023	76,195,710	76,195,710	73	66	239	212	13,189	10,694	2496	18.9
2024	77,045,898	77,045,898	72	65	236	205	13,171	10,356	2815	21.4
2025	77,892,526	77,892,526	72	65	234	199	13,150	10,023	3127	23.8
2026	78,735,596	78,735,596	72	64	231	192	13,127	9,696	3431	26.1
2027	79,575,106	79,575,106	72	64	229	185	13,102	9,373	3729	28.5
2028	80,411,057	80,411,057	72	63	226	178	13,075	9,056	4020	30.7
2029	81,243,448	81,243,448	72	63	224	171	13,047	8,742	4305	33.0
2030	82,072,280	82,072,280	72	62	221	164	13,016	8,431	4584	35.2
2017–2030							184.274	148,377	35,897	19.5

S2: CPR to increase from 63% in 2017 to 70% in 2030, and the unmet need to decrease from 10% in 2017 to 7% in 2030

**Table 7:** Estimated number of deaths averted, 2017–2030, under scenario 3

Year	WRA Base	WRA S3	GFR Base	GFR S3	MMR Base	MMR S3	MD Base	MD S3	MDA	% MDA
2017	71,019,837	71,019,837	73	73	255	255	13,251	13,251	-	-
2018	71,891,381	71,891,381	73	70	252	244	13,247	12,195	1053	7.9
2019	72,759,365	72,759,365	73	78	249	234	13,241	11,579	1662	12.6
2020	73,623,790	73,623,790	73	67	247	224	13,231	10,994	2238	16.9
2021	74,484,656	74,484,656	73	64	244	214	13,220	10,437	2782	21.0
2022	75,341,963	75,341,963	73	64	241	204	13,206	9,908	3298	25.0
2023	76,195,710	76,195,710	73	63	239	194	13,189	9,404	3786	28.7
2024	77,045,898	77,045,898	72	62	236	184	13,171	8,922	4249	32.3
2025	77,892,526	77,892,526	72	62	234	174	13,150	8,460	4690	35.7
2026	78,735,596	78,735,596	72	62	231	165	13,127	8,015	5112	38.9
2027	79,575,106	79,575,106	72	62	229	155	13,102	7,585	5517	42.1
2028	80,411,057	80,411,057	72	62	226	145	13,075	7,167	5908	45.2
2029	81,243,448	81,243,448	72	62	224	135	13,047	6,757	6289	48.2
2030	82,072,280	82,072,280	72	62	221	125	13,016	6,353	6663	51.2
2017–2030							184,274	131,027	53,247	28.9

S3: CPR to increase from 63% in 2017 to 75% in 2030, and the unmet need to decrease from 10% in 2017 to 5% in 2030

If CPR were to increase from 63% in 2017 to 70% in 2030 and UNFP to decline from 10 to 7%, it is estimated that 35,897 maternal deaths (19.5%) would be averted between 2017 and 2030 (Table 6). Sensitivity analyses involving assumptions regarding the responsiveness of fertility to declines in contraceptive use and of the MMR to declines in unmet need for family planning indicate that the impact could be as high as 37,186 maternal deaths averted (a 20% reduction) in the event of greater responsiveness and as low 34,621 maternal deaths averted (an 18.9% reduction) in the event of lower responsiveness. The estimates are thus rather robust to varying assumptions as to these underlying parameters, with estimates falling within a relatively narrow range of 34,000 to 37,000 maternal deaths averted.

Further optimizing the family planning program 2017–2030 by increasing CPR from 63 to 75% and decreasing UNFP from 10 to 5% would avert 53,247 maternal deaths (a 28.9% reduction) between 2017 and 2030 (Table 7). The range of estimates via sensitivity analysis performed as described above lie between 51,971 and 54,536 maternal deaths averted, reductions of 28.4% and 29.4%, respectively. The estimates again appear to be fairly robust to variations in assumptions concerning key underlying parameters.

## Discussion

The Indonesian national family planning program has over a period spanning just under 50 years (i.e., since 1970) which made a major contribution to the reduction of maternal mortality in the country. Had there not been any increase in the contraceptive prevalence rate from 1970 to 2017, it is estimated that there would have been at least 592,472 and as many as 663,000 additional maternal deaths during this period. This amounts to a 38–43% reduction. The maternal mortality rate, or the number of maternal deaths per 1000 women in a given year, fell by nearly 82% during this period. These estimates are plausible when compared with other global estimates [1, 7, 16].

In view of the success of national family planning efforts in achieving relatively high levels of contraceptive use, can family planning be expected to continue to make major future contributions to reducing maternal mortality? It is estimated that if the CPR were to be increased to 70% and unmet need for family planning reduced from 10 to 7% by 2030, an additional 35,897 (range 34,621–37,186) maternal deaths could be averted over and above the contributions of improvements in maternal health care in the country, a 19–20% reduction. If the CPR were instead to be increased to 75% and UNFP reduced to 5% by 2030, the number of additional maternal deaths averted would rise to 53,247 (range 51,971–54,536), a 28–29% reduction. However, even in the most optimistic scenario, there would still be over 13,000 maternal deaths in the year 2030, and the maternal mortality ratio would still be 125 maternal deaths per 100,000 live births, considerably above the global SDG target of less than 70 maternal deaths per 100,000 live births. Further advances in the provision of high-quality maternal health services by the health system to address risk once women become pregnant will be required in order to reach the SDG target.

Is achieving a CPR of 75%, or even 70%, by 2030 plausible? Such levels of contraceptive prevalence have already been achieved by Association of South-East Asian Nations (ASEAN) peer countries Thailand (78.4%) and Vietnam (76.7%) [27]. However, the growth in contraceptive use in Indonesia has been weak since the turn of the century, and some observers have pointed to a weakened state of the Indonesian national family planning program due to reduced international support, reduced government commitment, socio-political change, and weak local governance as important constraining factors [11]. Reaching a CPR of 70% in 2030 would require an annual CPR growth rate of 0.80% between 2018 and 2030. Reaching a CPR of 75% by 2030 would require an annual growth rate of 1.32%. By way of reference, the annual growth rate from 2000 to 2017 was 0.44%. Thus, the annual CPR growth rate would have to nearly double to reach the less ambitious target of 70% CPR in 2030, and triple to reach the more ambitious 75% target.

The slow growth in contraceptive prevalence in Indonesia in recent years is typical of countries with CPRs approaching or exceeding 60%. As demonstrated by Alkema et al. [2], the growth in contraceptive use in low- and middle-income countries has followed a logistic curve shaped like the letter “S”. This reflects the typical pattern of slow growth in contraceptive use following the introduction of family planning, which gives way to rapid growth once family planning programs have become established and are accepted by governments and sizeable proportions of national populations, followed by a period of slowing growth once the CPR reaches 50–60%. Indonesia is currently and has been since the mid-1990s/early 2000s at a level of contraceptive prevalence where gains have historically come slowly.

In such situations, priority attention needs to be directed to rectifying remaining geographic and socioeconomic inequities in access to accurate information and services, more effectively addressing local barriers and constraints to family planning uptake, providing informed choice across a full range of contraceptive methods, and improving service quality. Satisfying existing total demand for family planning, estimated to be 73.7% in the 2017 IDHS, would be sufficient to reach the 70% 2030 CPR target, but additional demand for family planning will be needed to reach the 75% 2030 CPR target.

In order to reinvigorate the national family planning program, meaningfully addressing age-, marital status-, and geography-related inequities in access to family planning services would be a logical place to start. At present, access to contraceptives by adolescents and unmarried women is restricted by law. With regard to geography, most Eastern Indonesia provinces and some provinces on the island of Sumatra have levels of demand for family planning and contraceptive use that lag the national norm, some provinces by a considerable margin [21]. Provinces in Eastern Indonesia also have less well-developed health infrastructure. Targeted, integrated family planning-maternal health initiatives that focus on locally-informed solutions to increasing demand for and use of contraception on the one hand and increased investment to address major health system supply-side readiness constraints on the other would seem to have considerable potential to achieve important results at both the local and national level.

Other issues also need to be addressed. The method mix of contraceptive use has shifted from the dominant use of long-acting, reversible contraceptives (IUDs and implants) to a decided dominance of short-term contraceptive methods (i.e., injectable contraceptives and pills) since the early 2000s [12]. Short-term contraceptives, due to higher discontinuation and failure rates, are less effective than long-term contraceptive methods [25] and lead to a larger number of unwanted pregnancies. Although the 2017 IDHS indicated a modest increase in the use of long-acting vs. short-term methods compared to the 2012 IDHS, the market share of traditional methods increased by an even greater amount [20]. Contraceptive discontinuation, that is the proportion of women who discontinue use within the first 12 months after initiation of a method, also remains high, especially for the two most popular contraceptive methods in Indonesia—oral contraceptives (46.1% market share) and injectables (27.7%) [20]. Although the 2017 IDHS data indicate that about 75% of women discontinuing use of oral contraceptives and injectables switch to a new method within six months, this method “churning” is suggestive of insufficient counseling and informed choice at the time of method adoption. Indeed, Indonesia fares rather poorly when it comes to the FP2020 core indicator “Method Information Index,” which measures the extent to which women were given specific information when they received family planning services in connection with adoption of the method they are currently using, with a 2017 “score” of only 34 on a 0-100 scale [24].

These challenges are formidable indeed. Some assistance in reaching the ambitious targets may come from the expansion of the national social health insurance scheme, the *Jaminan Kesehatan Nasional* (JKN). JKN population coverage reached 80% by the end of 2019, with the ultimate objective of achieving universal coverage. The JKN is likely to extend the reach of contraceptive services and supplies to areas that have limited private sector market penetration and make access to long-acting and permanent contraceptive methods more financially accessible.

## Conclusions

Substantial reductions in maternal mortality between the years 1970 and 2017 can be attributed to the successes of the national family planning program, and there are prospects for further contributions to the year 2030. However, the ability of Indonesia to reach the 2030 SDG maternal mortality target of 70 maternal deaths per 100,000 live births will depend more heavily upon the effectiveness of the health system in addressing health risks to women once they are pregnant.

## Data Availability

The bulk of the data used in the study were produced by the Government of Indonesia, Central Statistics Bureau (Badan Pusat Statistik - BPS), and are in the public domain. Please consult the BPS website for further information on publications and access to data—https://www.bps.go.id/. The Indonesian Demographic and Health Survey (IDHS) reports and data files used in the study are accessible via the DHS/IRC website: www.DHSprogram.com.

## Annex 1**.** List of Data Sources

Indonesian Demographic Health Surveys

Badan Pusat Statistik-Statistics Indonesia (BPS) and ORC Macro. (2003). ‘Indonesia Demographic and Health Survey 2002-2003’. Calverton, Maryland, USA: BPS and ORC Macro.

Central Bureau of Statistics (CBS) [Indonesia] and State Ministry of Population/National Family Planning Coordinating Board (NFPCB) and Ministry of Health (MOH) and Macro International Inc. (MI). (1988). ‘Indonesia Demographic and Health Survey 1987’. Calverton, Maryland: CBS and MI.

Central Bureau of Statistics (CBS) [Indonesia] and State Ministry of Population/National Family Planning Coordinating Board (NFPCB) and Ministry of Health (MOH) and Macro International Inc. (MI). (1992). ‘Indonesia Demographic and Health Survey 1991’. Calverton, Maryland: CBS and MI.

Central Bureau of Statistics (CBS) [Indonesia] and State Ministry of Population/National Family Planning Coordinating Board (NFPCB) and Ministry of Health (MOH) and Macro International Inc. (MI). (1995). ‘Indonesia Demographic and Health Survey 1994’. Calverton, Maryland: CBS and MI.

Central Bureau of Statistics (CBS) [Indonesia] and State Ministry of Population/National Family Planning Coordinating Board (NFPCB) and Ministry of Health (MOH) and Macro Intemational Inc. (MI). (1998). ‘Indonesia Demographic and Health Survey 1997’. Calverton, Maryland: CBS and MI.

Statistics Indonesia, National Family Planning Coordinating Board, Ministry of Health (MOH), and ORC Macro. (2003). ‘Indonesia Demographic and Health Survey 2002-2003’. Calverton, Maryland: CBS and MI.

Statistics Indonesia (Badan Pusat Statistik—BPS) and Macro International. (2008). ‘Indonesia Demographic and Health Survey 2007’. Calverton, Maryland, USA: BPS and Macro International.

Statistics Indonesia (Badan Pusat Statistik—BPS), National Population and Family Planning Board (BKKBN), and Kementerian Kesehatan (Kemenkes—MOH), and ICF International. (2013). ‘Indonesia Demographic and Health Survey 2012’. Jakarta, Indonesia: BPS, BKKBN, Kemenkes, and ICF International.

Indonesia Population Censuses and Intercensal Population Surveys

Statistics Indonesia (Badan Pusat Statistik—BPS). 1971. Population of Indonesia: Result of Indonesia Population Census 1971. Jakarta: Statistics Indonesia

Statistics Indonesia (Badan Pusat Statistik—BPS). 1980. Population of Indonesia: Result of Indonesia Population Census 1980. Jakarta: Statistics Indonesia

Statistics Indonesia (Badan Pusat Statistik—BPS). 1985. Population of Indonesia: Result of the 1985 Intercensal Population Survey. Jakarta: Statistics Indonesia

Statistics Indonesia (Badan Pusat Statistik—BPS). 1990. Population of Indonesia: Result of Indonesia Population Census 1990. Jakarta: Statistics Indonesia

Statistics Indonesia (Badan Pusat Statistik—BPS). 1995. Population of Indonesia: Result of the 1995 Intercensal Population Survey. Jakarta: Statistics Indonesia

Statistics Indonesia (Badan Pusat Statistik—BPS). 2000. Population of Indonesia: Result of Indonesia Population Census 2010. Jakarta: Statistics Indonesia

Statistics Indonesia (Badan Pusat Statistik—BPS). 2005. Population of Indonesia: Result of the 2015 Intercensal Population Survey. Jakarta: Statistics Indonesia

Statistics Indonesia (Badan Pusat Statistik—BPS). 2010. Population of Indonesia: Result of Indonesia Population Census 2010. Jakarta: Statistics Indonesia

Statistics Indonesia (Badan Pusat Statistik—BPS). 2015. Population of Indonesia: Result of the 2015 Intercensal Population Survey. Jakarta: Statistics Indonesia

**Other Reports/documents**

Hogan M.C., Foreman K.J., Naghavi M., Ahn S.Y., Wang M., Makela S.M., Lopez A.D, Lozano R., and Murray C.J. (2010). ‘Maternal mortality for 181 countries, 1980-2008: a systematic analysis of progress towards Millennium Development Goal 5’. Lancet, 375, 9726: 1609 – 1623.

IBFAN (2014). ‘Report on the Situation of Infant and Young Child Feeding in Indonesia’. Jakarta: Indonesian Breastfeeding Mothers Association.

Iskandar M.B., Utomo B., Hull T., Dharmaputra N.G., and Azwar Y. (1996). ‘Unraveling the mysteries of maternal death in West Java: Reexamining the witnesses. [1st ed.]’. Depok: Center for Health Research, Research Institute, University of Indonesia.

National Research Council. (2013). ‘Reducing Maternal and Neonatal Mortality in Indonesia: Saving Lives, Saving the Future’. Washington, DC: The National Academies Press

Statistics Indonesia (Badan Pusat Statistik-BPS) and National Family Planning Coordinating Board. (1987). ‘Indonesia- National Contraceptive Prevelance Survey 1987’. Calverton, Maryland: CBS and MI.

Statistics Indonesia (Badan Pusat Statistik-BPS), National Family Planning Coordinating Board, Ministry of Health and ICF. (2018). Survei Demografi and Kesehatan Indonesia 2017: Laporan Pendahuluan Indikator Utama. Jakarta: Statistics Indonesia.

Statistics Indonesia (Badan Pusat Statistik-BPS), Bappenas and UNFPA. (2013). ‘Indonesia Population Projection 2010-2035’. Jakarta: Statistics Indonesia.

UNDP (2004). ‘Human Development Report 2004, Cultural Liberty in Today’s Diverse World’. New York: UNDP.

Utomo B. (1976). ‘A study of Indonesian Age Data, 1971 and 1976’. Jakarta: Lembaga Demografi FEUI.

## Abbreviations

ASFR: Age-specific fertility rate
CPR: Contraceptive prevalence rate
FP2020: Family Planning 2020
GFR: General fertility rate
IDHS: Indonesia Demographic and Health Survey
JKN: Jaminan Kesehatan Nasional
MD: Maternal deaths
MDA: Maternal deaths averted
MMR: Maternal mortality rate
SDG: Sustainable Development Goals
TFR: Total fertility rate
WRA: Women of reproductive age
